# Small or far away? Size and distance perception in the praying mantis

**DOI:** 10.1098/rstb.2015.0262

**Published:** 2016-06-19

**Authors:** Vivek Nityananda, Geoffrey Bissianna, Ghaith Tarawneh, Jenny Read

**Affiliations:** 1 Institute of Neuroscience, Henry Wellcome Building for Neuroecology, Newcastle University, Framlington Place, Newcastle upon Tyne NE2 4HH, UK; 2 M2 Comportement Animal et Humain, École doctorale de Rennes, Vie Agro Santé, University of Rennes 1, Rennes 35000, France

**Keywords:** size constancy, stereo vision, *Sphodromantis lineola*

## Abstract

Stereo or ‘3D’ vision is an important but costly process seen in several evolutionarily distinct lineages including primates, birds and insects. Many selective advantages could have led to the evolution of stereo vision, including range finding, camouflage breaking and estimation of object size. In this paper, we investigate the possibility that stereo vision enables praying mantises to estimate the size of prey by using a combination of disparity cues and angular size cues. We used a recently developed insect 3D cinema paradigm to present mantises with virtual prey having differing disparity and angular size cues. We predicted that if they were able to use these cues to gauge the absolute size of objects, we should see evidence for size constancy where they would strike preferentially at prey of a particular physical size, across a range of simulated distances. We found that mantises struck most often when disparity cues implied a prey distance of 2.5 cm; increasing the implied distance caused a significant reduction in the number of strikes. We, however, found no evidence for size constancy. There was a significant interaction effect of the simulated distance and angular size on the number of strikes made by the mantis but this was not in the direction predicted by size constancy. This indicates that mantises do not use their stereo vision to estimate object size. We conclude that other selective advantages, not size constancy, have driven the evolution of stereo vision in the praying mantis.

This article is part of the themed issue ‘Vision in our three-dimensional world’.

## 1. Introduction

Stereo vision is a remarkable computational capability. It uses complex algorithms to take advantage of the disparity between the views of the world seen by each eye [1–4]. This is a costly process with its own dedicated neural matter and computational power [3,4]. It has nonetheless evolved to be a specialized perceptual capacity in humans and in other animals including owls [5], horses [6] and insects [7]. It appears, furthermore, to have evolved independently in at least three evolutionary lineages [7,8]. This suggests that there must be large selective advantages to stereo vision that benefit the animals in which it has evolved.

What possible advantages could stereo vision confer? The binocular disparities detected by a stereo vision system depend on the distance from the eyes to the object. Stereo vision is therefore profoundly related to distance. In primates, this relationship is complicated by our highly mobile eyes, which means there is no fixed mapping from binocular disparity to distance. Probably for this reason, we are better at discriminating the relative depth between adjacent objects rather than the absolute distance to an object [2]. Critically, we can still detect this relative depth boundary even if the object in question would otherwise be perfectly camouflaged against the background. A key advantage of stereo vision may therefore be that it confers the ability to detect camouflaged objects [9]. We know that humans, monkeys and owls can all use their stereo vision in this way [9–11]. This kind of ‘camouflage breaking’ could be an important evolutionary advantage—think of a predator spotting prey against a similar-looking background. A related advantage of binocular, if not strictly stereoscopic, vision may be that it helps animals see more of the background behind an object, enabling a degree of ‘X-ray’ vision [12]. This could help an animal spot a predator hidden behind vegetation clutter.

In animals where the eyes are fixed in the head, like insects, or nearly so, like owls, stereo vision may be equally important for judging the absolute distance to an object. This would be useful to an owl trying to catch prey or a praying mantis reaching for a fly at particular depth, and we know that mantises do indeed use absolute disparity information in this way [7,13]. There is further evidence that mantises might be sensitive to prey at different distances even within their catch range and adjust their strikes accordingly [14]. This is similar to how toads, with very low ocular mobility, adjust their tongue extensions to capture prey based on distance information provided by absolute disparity cues [15]. It has been suggested that stereo vision in mantises is specialized for this range-finding function and is thus possibly simpler than primate stereo [16].

Information about absolute distance could also be used to calibrate other cues. For example, disparity cues in combination with angular size could allow animals to unambiguously judge the physical (as opposed to apparent) size of objects [17], distinguishing between a small object that is nearby or a large object that is far away. This could be advantageous if, for example, a predator needed to catch prey of a particular size. It could also make all the difference for an organism trying to decide whether an object is a small prey animal (and could be captured) or a large predator (and requires defensive action).

It should be noted, however, that cues unrelated to disparity could also help an organism judge both absolute and relative depth. These include motion parallax, shading, focus blur and relative object size. Humans make use of these cues [18] and under appropriate circumstances these can be more useful for depth perception than disparity [19]. Other animals also make use of similar cues to tell depth. Mantises, for example, make use of motion parallax to judge the width of gaps they need to jump across [20].

There are thus several possible advantages to stereo vision, but which of these advantages leads to the evolution of stereo vision might differ in each animal. Each of the advantages listed above would be important only in the context of the specific ecology of each species. Animals in denser habitats might have a greater need for X-ray vision; predators whose prey has evolved background-matching coloration might have a greater need for camouflage breaking; while predators that specialize on specific prey might need to judge object size and distance more accurately. It is important therefore to explore the advantages to each animal known to have stereo vision in relation to their ecology. In this paper, we investigate whether praying mantises use their stereo vision to help judge prey size as well as distance.

Praying mantises are specialized visual predators with a high degree of binocular visual overlap (35° in *Tenodera australiae* [21]). Many species of mantises capture prey by sitting motionless until prey passes by within their catch range [22]. They then reach out with a rapid reaching motion of their forelegs, called a strike, and capture their prey [23]. Stereo vision is thus a big advantage to them and early experiments indicated that they were capable of using binocular cues to judge depth [24]. Stereopsis in praying mantises was first demonstrated by placing prisms in front of mantis's eyes and bringing a fly closer to the mantis [7]. As mantises typically strike only when the approaching prey is perceived to be in the correct catching range (around 2.5–5 cm for several species) [23], the strikes are a good measure of their judgements of depth. These experiments showed that mantises were striking based not on absolute distance to the fly but on disparity cues that were manipulated using the prisms. Apart from these early experiments, however, we know little about the mechanisms of mantis stereopsis and what advantages it might confer to mantises.

**Figure 1.**
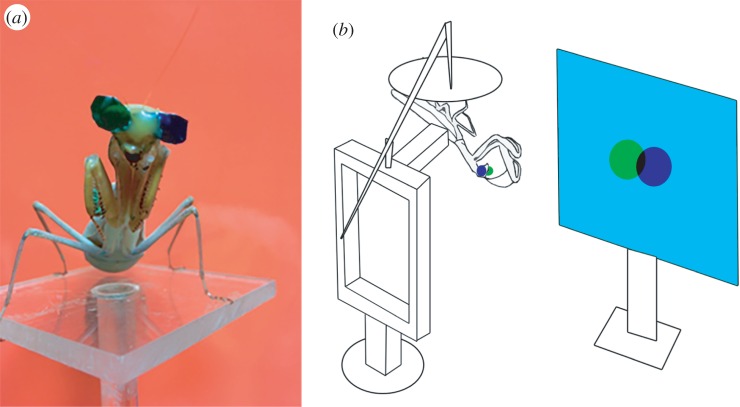
Insect 3D cinema. (*a*) Mantises were fitted with green-blue colour glasses. (*b*) 3D virtual targets were presented to the mantises in a 3D insect cinema. (*b*) Reproduced with permission from Nityananda *et al.* [13]. (Online version in colour.)

One of the barriers to further investigation of mantis stereopsis has been the lack of experimental paradigms using 3D virtual stimuli that have revolutionized the study of stereo vision in primates. We, therefore, recently developed an insect cinema where we used anaglyph technology with mantises wearing blue and green filters on their eyes (figure 1*a*) to show mantises virtual 3D stimuli [13]. Using this set-up, we definitively demonstrated stereopsis in mantises and opened up the possibility of further investigations into mantis stereo vision. In this paper, we use this 3D insect cinema to explore how mantises use disparity and angular size cues to assess the size of objects and make their decisions to make predatory strikes. We were especially interested to see if mantises show size constancy, the phenomenon where an organism combines depth information and image size to compute an object's physical size [17,25]. If they specialized on a particular size of prey, we would expect them to be able to respond selectively to combinations of disparity and angular size cues that corresponded to a specific absolute size of prey (table 1).

**Table 1.** Simulated sizes (cm) for every combination of simulated distances and prey angular sizes presented to the mantises. The entries in italics are an example of the expected pattern of conditions at which the mantises would strike maximally if they struck at prey of a specific absolute size, i.e. if they displayed size constancy. In our example, their most preferred absolute size is 0.74 cm.

		angular size (°)			
		7.5	11.25	16.88	25.31
simulated distance (cm)	2.5	0.33	0.49	*0.74*	1.11
	3.75	0.49	*0.74*	1.11	1.68
	5.63	*0.74*	1.11	1.68	2.53

Size constancy would appear to be important for praying mantises given their behavioural ecology. Mantises will catch and eat insects such as locusts, but sufficiently large locusts are entirely capable of eating the mantis instead. Mantises are also predated by larger species such as birds. It might well be important, therefore, for mantises to avoid striking at prey that was too large.

An early study comparing deimatic responses (threat displays) of monocular and binocular mantises showed that monocular mantises responded to similar angular sizes of birds while binocular mantises responded based on the distance cues to the birds [26]. This suggested that binocular mantises could perhaps take distance into account when responding to differently sized objects. Another study specifically investigated size constancy to prey objects using prisms to manipulate the disparity cues available to the mantis independently of the size of the stimulus presented [14]. This study found no evidence for size constancy in the mantis; mantises struck at stimuli of a constant angular size. This is a surprising result given the potential value to the mantis of knowing the true size of prey. One possibility is that mantises were able to view the prey stimulus at its true distance, without disparity manipulated by the prisms, when it first appeared in the periphery of the display screen. Another possible factor is that prisms move the entire visual scene, not just the target, nearer to the mantis, which might influence its striking behaviour. Our methods would be able to prevent both these problems. As our 3D glasses are fixed to the head, the mantis fovea would always view the stimuli with the intended disparity cues, and these cues would apply only to the simulated prey item while the rest of the visual scene would present constant, veridical cues. We therefore revisited this important question with our completely different stereoscopic display technology to test whether we could find evidence for size constancy in the praying mantis.

## 2. Methods

### (a) Experimental subjects

We carried out all experiments on female mantises of the species *Sphodromantis lineola*. We housed the mantises in individual plastic boxes (7 cm length × 7 cm breadth × 9 cm height) with holes in their lids to allow for ventilation. The mantises could move freely within the boxes. The boxes were stored in a housing facility, which we maintained at 25°C. We cleaned the boxes, misted them with water, and fed each mantis a live cricket twice a week.

### (b) Stimuli and display

We used a DELL U2413 LED monitor to display the stimuli to the mantis. This monitor has narrowband spectral output in the blue and green regions of the spectrum and we have previously shown that it is effective at producing an illusion of 3D to the mantises in conjunction with the anaglyph glasses we used [13]. The monitor has a resolution of 1920 × 1200 pixels and a 60 Hz refresh rate and is 51.8 cm wide by 32.4 cm high. All stimuli were custom written in Matlab (Mathworks) using the Psychophysics Toolbox. All stimulus presentations consisted of a dark swirling disc against a uniform bright background that spiralled in from the periphery to the centre of the screen in 5 s (for further details of the stimulus and the display, see Nityananda *et al.* [13]). The disc had an angular position *θ*(*t*) = 4*πt* and a radial position *r*(*t*) = 10(1 + cos(min(*tπ*/5,*π*))) cm. The disc thus spiralled in from a distance of 20 cm towards the centre of the screen, with smooth initial acceleration and final deceleration, over a duration of 5 s. At the centre of the screen, the disc moved with subtle jerky motions for a further 2 s and then vanished. This stimulus reliably elicits strikes when presented with a diameter of 1 or 2 cm and zero disparity, with the screen being 2.5 cm from the mantis, i.e. within the catching range.

We should note that light from LED monitors is linearly polarized, and several insects are known to be sensitive to linear polarization. However, this polarization would apply equally to all stimuli presented on the screen and would not affect the illusory perception of depth generated by the use of anaglyph glasses.

### (c) Preparation and fixation of the three-dimensional glasses

To be able to present the mantis with different disparity cues, we fitted each mantis with green and blue glasses (figure 1*a*). These glasses were teardrop shaped with a maximum length of around 7 mm and cut out of filters distributed with a preprint of a previously published paper [27]. We have previously shown that these filters have very low spectral overlap and are effective in conveying an illusion of 3D to the mantises [13].

Before fixing the glasses, we placed the entire cage in which the mantis was housed in a freezer (Argos Value Range DD1-05 Tabletop Freezer) for 5–7 min to immobilize it. We then took the mantis out and held it down under a microscope using Plasticine^®^ modelling clay (Flair Leisure Products plc). We fixed the glasses onto the mantis using a mixture of beeswax and rosin, which we melted and applied using a Denta Star S ST 08 wax melter. The assignment of the blue and green glasses to the left and right eyes was counterbalanced across all insects used in the study. We also fixed a small component, designed for electronics, onto the base of the mantis's pronotum. This fit into a counterpart on the experimental stand and held the mantis in place during experiments while leaving the movement of the head and forelimbs unrestricted. After fixing the glasses and the component, we released the mantises and placed them in their cages. We gave them at least 24 h to recover before we carried out any experiments.

### (d) Experimental set-up

We fixed the mantis onto a stand using the component attached to its pronotum. We positioned the mantis upside down, a position mantises are comfortable with while hunting, and provided them with a cardboard disc that it held onto for stability (figure 1*b*). We placed the stand so that the distance between the mantis and the screen was 10 cm. The stand was the one used by Rossel [7] in his earlier experiments investigating stereo vision.

### (e) Experimental protocol

**Figure 2.**
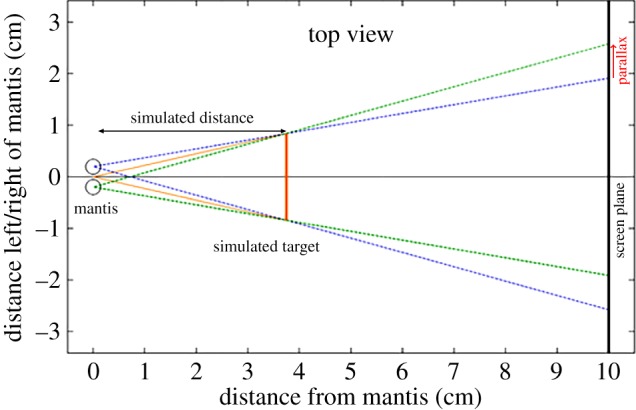
Top-down view showing how presenting stimuli with on-screen parallax simulates an object in front of the screen. The blue and green dashed lines show how to compute the image position in order to simulate a disc at 3.75 cm in front of the mantis. We use the term parallax to refer to the difference in on-screen position between left and right images. (Online version in colour.)

We presented the stimuli to each mantis in several runs during which we varied the disparity and angular size of the disc stimulus (figure 2). We used disparity to present virtual stimuli at different simulated distances from the mantis (figure 2); the physical distance of the stimuli was always the same (i.e. the distance of the screen, 10 cm from the mantis). Each combination of simulated distance and angular size corresponded to a specific simulated object size (table 1). Each run consisted of 24 trials encompassing four different angular sizes of the disc each presented in six different disparity conditions. The trials were presented in random order with a pause of 60 s between each trial. The four angular sizes used were 7.49°, 11.25°, 16.87° and 25.31°. Three of the six disparity conditions were ‘crossed disparity’ conditions where we presented the image visible to each eye contralateral to that eye, so that the lines of sight from the two eyes crossed in front of the screen (figure 2). In these conditions, we presented targets at simulated distances of 2.5 cm, 3.75 cm and 5.63 cm from the mantis. All these distances are approximately within the catch range of the mantis [23]. Assuming an interocular distance of 0.7 cm, these corresponded to parallaxes (the physical separations between the left and right images on the screen; figure 2) of 2.1 cm, 1.16 cm and 0.54 cm, respectively. The other three conditions were control conditions where we presented stimuli with the same parallax on the screen as the first three but with the left and right images swapped, i.e. ipsilateral to the eyes that could view them. These conditions presented the mantis with stimuli where the left and right eye images failed to converge. They cannot be interpreted as images of a single object, let alone one within the catch range, and we therefore expected them to be unattractive to the mantis. We tested six mantises with ten experimental runs of 24 trials each and one more mantis with six experimental runs.

### (f) Data recording and analysis

For every presentation of a stimulus, we recorded the mantis's response using a Kinobo USB B3 HD Webcam (Point Set 248 Digital Ltd, Edinburgh, UK) camera placed underneath the mantis. The camera did not have a view of the monitor and all recordings were thus blind to the stimulus condition. We analysed the recorded videos manually. For each trial, we noted the number of times the mantis made predatory strikes with its forelegs as well as the times it moved its head to track the stimulus (referred to as ‘strikes’ and ‘tracks’ below). The parameters of the stimulus corresponding to each stimulus presentation were saved by the computer and, after the videos were analysed, we matched the recorded number of strikes with the corresponding stimulus parameters.

To see if there were significant main effects of both simulated distance and angular size on the number of strikes made by the mantises, we ran a generalized linear model (GLM) with the number of strikes for each individual presentation as the dependent variable. As this involved count data, we assumed a Poisson distribution with a log-linear link function. We used the identity of the animal, the simulated distance and the angular size as factors in the model. We used the model to investigate a main effect of the simulated distance, the angular size and an interaction effect between the simulated distance and angular size. We ran separate models for the crossed and uncrossed disparity presentations. We also ran models for each of these conditions using the absolute size in mm instead of angular size as a factor. Finally, we also ran models with the number of tracks in individual trials as a dependent variable.

To assess if the simulated distance and the angular size had independent effects on the number of strikes made by the mantis, we ran a *χ*^2^-test. We next assessed if the mantises preferred a particular simulated distance after accounting for the main effect of angular size. To do this, we normalized the number of strikes made by each individual for every simulated distance by the maximum number of strikes made by that individual in response to any angular size for that distance. We then ran a two-way Friedman's ANOVA to see if there was a significant effect of simulated distance and angular size on the normalized number of strikes. If there was a preferred distance regardless of angular size, we should then expect to find a significant effect of the distance but not the angular size on the normalized number of strikes.

To assess evidence for size constancy, we normalized the number of strikes made by each individual for every angular size by the maximum number of strikes made by that individual in response to any simulated distance for that angular size. We then ran a two-way Friedman's ANOVA to see if there was a significant effect of simulated distance and angular size on the normalized number of strikes. If mantises showed a preferred physical size independent of distance, we should then expect to find a significant effect of both the distance and the angular size on the normalized number of strikes. A fixed physical size preference would also further predict that the number of strikes would be greater for larger angular sizes at closer distances, and for smaller angular sizes at farther distances.

## 3. Results

**Figure 3.**
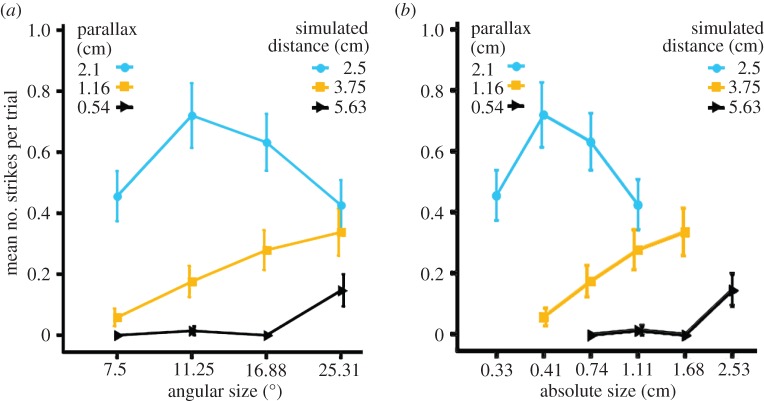
Behavioural response of mantises in the crossed disparity condition. Mean number of strikes in response to different parallaxes (and the corresponding simulated distances) plotted as a function of (*a*) the angular size of the simulated target and (*b*) the absolute size of the simulated object. Error bars indicate standard error. Overlapping bars have been staggered so they can be viewed clearly. (Online version in colour.)

In the crossed disparity trials, screen parallax simulated targets in front of the screen. We found a significant main effect of both simulated distance (GLM: Likelihood ratio 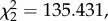 *p* < 0.001) and angular size (GLM: Likelihood ratio 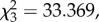 *p* < 0.001) on the number of strikes made during a presentation (figure 3*a*). There was also a significant interaction effect between simulated distance and angular size (GLM: Likelihood ratio 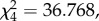 *p* < 0.001) on the number of strikes made during a presentation (figure 3*a*). We also confirmed that simulated distance and angular size did not have independent effects on the number of strikes made (*χ*^2^-test, 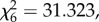 *p* < 0.001). We found similar results when we used the simulated absolute size as a predictor of the number of strikes rather than angular size (figure 3*b*).

We also saw a significant main effect of the angular size (GLM: Likelihood ratio 
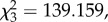
 *p* < 0.001) and simulated distance (GLM: Likelihood ratio 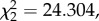 *p* < 0.001) on the number of tracks made during a presentation. The interaction effect of simulated distance and angular size on the number of tracks made was not significant (GLM: Likelihood ratio 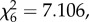 *p* = 0.311). Having shown that both simulated distance and angular size have a significant effect on strike rate, we then asked whether mantises show a consistent preference for a given distance or size.

### (a) Mantises have a clear distance preference

**Figure 4.**
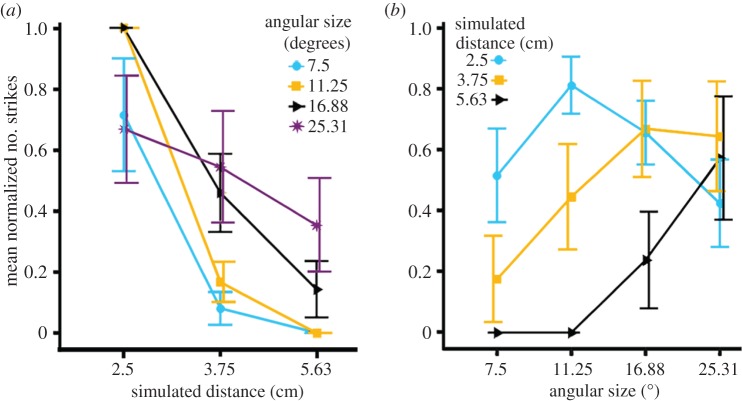
Normalized behavioural responses of the mantises in the crossed disparity condition. Mean normalized number of strikes in response to different angular sizes and simulated distances. Strikes were normalized by (*a*) the maximum number of strikes to any angular size for a given simulated distance and (*b*) the maximum number of strikes to any simulated distance for a given angular size. See text for details. Overlapping bars have been staggered so they can be viewed clearly. (Online version in colour.)

Whether we examine tracks or strikes, the mantises show a clear preference for targets at a simulated distance of 2.5 cm (blue circles in figure 3), rather than 3.75 cm or 5.63 cm (orange squares, black triangles). We asked if there was a preference for a simulated distance after controlling for the main effect of angular size. We normalized the number of strikes for every simulated distance by the maximum number of strikes for any angular size for that distance. We found a significant effect of simulated distance on the normalized number of strikes (figure 4*a*, Friedman's two-way ANOVA 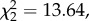 *p* < 0.01) but not of angular size (figure 4*a*, Friedman's two-way ANOVA 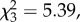 *p* = 0.145). This indicates that mantises do not prefer all simulated distances equally even after we control for angular size effects through normalization.

**Figure 5.**
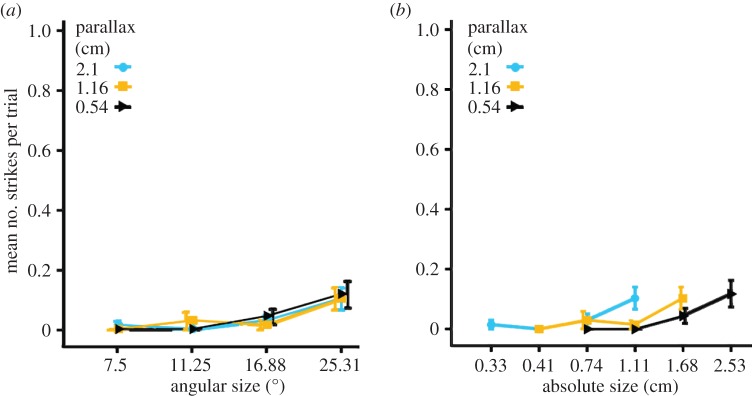
Behavioural response of mantises in the uncrossed disparity condition. Mean number of strikes in response to different parallaxes as a function of (*a*) the angular size of the simulated target and (*b*) the absolute size of the simulated object. Error bars indicate standard error. Overlapping bars have been staggered so they can be viewed clearly. (Online version in colour.)

We can be confident that this preference is indeed driven by the distance simulated by the parallax, rather than some other aspect of the stimulus, by comparing results in the uncrossed control condition (figure 5*a,b*). As expected, the response rates in the uncrossed disparity condition were much lower than those seen for crossed disparity (figure 5*a,b*). In addition, in this condition there was no significant main effect of the simulated distance (GLM: Likelihood ratio 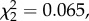 *p* = 0.968) on the number of strikes in each presentation. There was also no significant interaction effect between simulated distance and angular size (GLM: Likelihood ratio 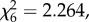 *p* = 0.894) on the number of strikes in each presentation. Angular size, however, did have a significant main effect (GLM: Likelihood ratio 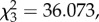 *p* < 0.001). The results in the uncrossed condition confirm that the effect of parallax in the crossed condition was due to the simulation of near distance, as intended. If, say, the mantis visual system simply summed images from the two eyes and then struck preferentially at the larger combined image associated with larger disparity, then we would have seen the same effect for both crossed and uncrossed conditions.

Our results therefore show that mantises have a strong preference for prey at a distance of 2.5 cm as compared to prey that is further away, when these distances are indicated solely by binocular disparity. The ordering of the distance preference is not affected by the angular size of the prey, although the strength of the preference may be.

### (b) Mantises show no consistent size preference

We now turn to the critical question of size constancy. In contrast with distance, we found that angular size preferences are not consistent. At the closest simulated distance of 2.5 cm, the mean number of strikes was highest for a prey angular size of 11.25° but for simulated distances of 3.75 or 5.63 cm this shifted to 25.31° (figure 3*a* and table 2). We examined if there was a preference for any angular size after controlling for the main effect of distance by normalizing the number of strikes for every angular size by the maximum number of strikes for any simulated distance for that angular size. We found a significant effect of both simulated distance (figure 5*b*, Friedman's two-way ANOVA 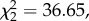 *p* < 0.01) and angular size (figure 4*b*, Friedman's two-way ANOVA 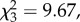 *p* = 0.02) on the number of normalized strikes. This suggests that even after the main effect of simulated distance is controlled for, we still have an interaction effect between simulated distance and angular size with different preferences for angular size depending on the simulated distance. This interaction is, however, not in the direction that one would expect if the mantises had size constancy. The mantises thus did not prefer any specific object size independent of simulated distance.

**Table 2.** Mean number of strikes per trial, for every combination of simulated distance and prey angular size presented to the mantises. The highest mean number for every simulated distance is marked in italics. The pattern fails to follow that indicated by size constancy as indicated in table 1.

		angular size (°)			
		7.5	11.25	16.88	25.31
simulated distance (cm)	2.5	0.47	*0.72*	0.62	0.41
	3.75	0.07	0.17	0.28	*0.34*
	5.63	0.00	0.00	0.05	*0.15*

## 4. Discussion

We used our ‘insect 3D cinema’ to investigate the influence of binocular disparity and angular size cues on mantises’ decisions to strike at prey. We know that mantises use both size and distance in deciding whether to strike at potential prey, and we know that they can judge distance from binocular disparity alone. We were interested in whether mantises use the distance information provided by disparity to calibrate angular size in order to perceive the correct physical size of objects over a range of distances. This correct perception is known as size constancy [17,25]. To examine this, we tested whether mantises strike selectively at prey that is both at a particular distance and of a particular physical size. This would require their preference for angular size to depend on the target distance.

We found that disparity had an overall influence on the number of strikes made by the mantises and that angular size did indeed influence the mean number of strikes differently for different disparities. Mantises struck most often for targets whose disparity indicated they were 2.5 cm from the animal, and made fewer strikes for disparities that indicated distances of 3.75 and 5.63 cm, confirming that disparity cues clearly influence their decisions to strike. This confirms Rossel's [14] conclusion that mantises can use binocular disparities to discriminate objects at 3.5, 4.5 and 5.5 cm. This is certainly what we would expect based on the optics. The angle *α* subtended by the mantid's eye separation *I* at a distance *D* is given by *I* = 2*D* tan(*α*/2). For an interocular distance of 0.7 cm, the difference in α for a target at 4.5 versus 5.5 cm is 1.6^o^, much larger than the interommatidial separation at the fovea, around 0.5^o^ [21]. The minimum discriminable distance *δD* depends on the baseline distance *D*: *δD* = *δα* (4*D*^2^ + *I*^2^)/4*I*. If we make the conservative assumption that the smallest detectable disparity change *δα* is the interommatidial separation, 0.5^o^, we predict that mantises should be able to discriminate distances of 3 mm at 5 cm, or 1 cm at 10 cm, or 5 cm at 20 cm. Distances > 80 cm would be indistinguishable from infinity.

The preference for distance was consistent, independent of object size (figure 4*a*). By contrast, mantises displayed no consistent preference either for angular or physical size (figures 4*b* and 3*b*). Mantises struck at an angular size of 11.25° for the closest simulated distance and at the highest angular size of 25.31° for the higher simulated distances, i.e. the preferred angular size varied with distance. However, the variation was not consistent with a single preferred physical size (figure 3*b*). A consistent preference for prey of a particular physical size would predict that mantises should strike at greater angular sizes of prey perceived to be close, and smaller angular sizes of prey perceived to be farther away. Instead, we found that mantises struck at smaller angular sizes for the closest simulated distance, and at the highest angular sizes for the higher simulated distances (figures 3 and 4*b*).

A previous study [14] of the influence of distance on size estimation in the praying mantis used prisms and objects presented on a TV screen to address a similar question. This study also showed that mantises do not consistently prefer prey of a given physical size, and argued that the angular size predominantly drives their prey catching behaviour. Our results differ from the results of this study. In our study, mantises do not consistently prefer prey of a given angular size: their preferred angular size reduces for closer prey. The previous study examined angular sizes from 15° to 60°; it did not test mantises at the lower angular sizes we did and it is possible that this is why it did not see the effect we did. It also used a different species of *Sphodromantis* (*S. viridis* rather than *S. lineola*), and we noted some further, potentially important methodological differences in the Introduction. Our results show that in *S. lineola*, disparity-defined distance does alter the preference for angular size.

It is possible that mantises do use their stereo vision to deduce true physical size—i.e. that they have size constancy—but that their preference for prey physical size genuinely varies with distance. For example, capturing prey near the limit of their catch range could be more energetically expensive. They might therefore only strike out at prey that is farther away when it also appears to be bigger and therefore worth the energetic cost. Alternatively, the way the mantis' forelegs unfold during the strike might make it more difficult to capture larger prey that is nearby, compared with smaller prey. Rossel [14] found, for example, that at shorter distances the femur impacts on prey from above, while at longer distances it impacts from below.

It is also possible that mantis stereopsis, and thus size constancy, works only over a limited range. For example, at farther distances, mantises might rely mainly on angular size to judge prey size, while at nearer distances they use the combination of disparity and angular size. It is interesting to compare the crossed and uncrossed disparity conditions with this idea in mind. The peak number of strikes in the uncrossed disparity conditions is the same as that for the farther simulated distances in the crossed disparity conditions: both occurred at an angular size of 25.31°. This might perhaps argue that when disparity cues are ambiguous or do not indicate nearby objects, mantises default to using angular size as the cue on which to base their decisions to strike.

Size estimation has been studied in other insects [14,28] and there has so far been no clear indication of size constancy in insects. Some studies have suggested that dragonflies do not use angular size alone to estimate prey size [28] and our data would also support this idea in mantises, even though they do not show any evidence for size constancy. In the previous study of size estimation and its dependency on distance in mantises [14], the author found results similar to ours showing that distance influenced the probability of striking in mantises. As we found in our study, he, however, also showed that there is no preference for an absolute (mm) size. It might be possible that size constancy matters in a different context—one of distinguishing between predators and prey [26], which involves larger disparities than the ones we have presented in our experiment. It might also be interesting to examine size constancy in different species of praying mantises. The species we tested, S*. lineola*, appears to be quite generalist in its choice of prey and this might explain why we fail to see any evidence of size constancy in this species. Other species that specialize on specific prey might show more evidence for size constancy.

Our study provides no evidence that mantises can use binocular disparity to compute the absolute size of prey. Stereo vision nonetheless has major advantages for the mantis. It definitely helps the mantis judge whether prey is at a depth within capture range or not, as indicated by the clear preference for near distances simulated only by disparity. While mantises can also use motion parallax for depth judgements, they appear to use this more for judging the gaps they might need to jump over [29]. Furthermore, using motion parallax would require them to move. This would give their position away to prey and would thus work against their predatory strategy. Stereo vision thus enables them to judge prey distance without moving and to strike only when prey is at the right depth. Another possible selective advantage is that stereo vision might enable mantises to spot a camouflaged object, similar to primates and owls. This is an intriguing possibility and has not yet been tested. Further work thus remains to be done to fully understand the evolution of stereo vision in insects and how its mechanisms differ from those in primates and other animals.

## Supplementary Material

Mantis strikes and tracks in response to different disparity and angular size conditions

## Data accessibility

The datasets supporting this article have been uploaded as part of the electronic supplementary material.
